# Valine metabolites analysis in ECHS1 deficiency

**DOI:** 10.1016/j.ymgmr.2021.100809

**Published:** 2021-10-09

**Authors:** Mari Kuwajima, Karin Kojima, Hitoshi Osaka, Yusuke Hamada, Eriko Jimbo, Miyuki Watanabe, Shiho Aoki, Ikuko Sato-Shirai, Keiko Ichimoto, Takuya Fushimi, Kei Murayama, Akira Ohtake, Masakazu Kohda, Yoshihito Kishita, Yukiko Yatsuka, Shumpei Uchino, Masakazu Mimaki, Noriko Miyake, Naomichi Matsumoto, Yasushi Okazaki, Tomomi Ogata, Takanori Yamagata, Kazuhiro Muramatsu

**Affiliations:** a Department of Pediatrics, Jichi Medical University, Tochigi, Japan; b Department of Pediatrics, Toyonaka Municipal Hospital, Osaka, Japan; c Department of Pediatrics, Tokyo Metropolitan Fuchu Ryoiku Center, Tokyo, Japan; d Department of Metabolism, Chiba Children's Hospital, Chiba, Japan.; e Department of Pediatrics & Clinical Genomics, Faculty of Medicine, Saitama Medical University, Saitama, Japan; f Center for Intractable Diseases, Saitama Medical University Hospital, Saitama, Japan; g Diagnostics and Therapeutics of Intractable Diseases, Intractable Disease Research Center, Graduate School of Medicine, Juntendo University, Tokyo, Japan; h Department of Life Science, Faculty of Science and Engineering, Kindai University, Osaka, Japan.; i Department of Pediatrics, Teikyo University School of Medicine, Tokyo, Japan.; j Department of Human Genetics, Yokohama City University Graduate School of Medicine, Yokohama, Japan; k Department of Human Genetics, Research Institute, National Center for Global Health and Medicine, Tokyo, Japan.; l Laboratory for Comprehensive Genomic Analysis, RIKEN Center for Integrative Medical Sciences, Kanagawa, Japan; m Department of Pediatrics, Gunma University Graduate School of medicine, Maebashi, Japan

**Keywords:** Short-chain enoyl-CoA hydratase deficiency, Leigh syndrome, Diet therapy, ECHS1, short-chain enoyl-CoA hydratase, LC-MS/MS, liquid chromatography with tandem mass spectrometry, SCPC, S-(2-carboxypropyl) cysteine, SCPCM, S-(2-carboxypropyl) cysteamine, SCEC, S-(2-carboxyethyl)cysteine, SCECM, S-(2-carboxyethyl)cysteamine

## Abstract

Short-chain enoyl-CoA hydratase (ECHS1) is involved in amino acid and fatty acid catabolism in mitochondria and its deficiency causes Leigh syndrome or exercise-induced dystonia. More than 60 patients with this condition have been reported till date. The accumulation of intermediate metabolites of valine is assumed to be responsible for the cytotoxicity. Since protein restriction, including valine reportedly improves neurological symptoms, it is essential to consider the possible incidence of and diagnose ECHS1 syndrome in the earlier stages. This study reported the liquid chromatography with tandem mass spectrometry (LC-MS/MS) urine and plasma metabolite analysis in six cases, including four new cases with ECHS1 deficiency. The values of urine cysteine/cysteamine conjugates from valine metabolites, S-(2-carboxypropyl) cysteine/cysteamine from methacrylyl-CoA, and S-(2-carboxyethyl) cysteine/cysteamine from acryloyl-CoA were separated between six patients and six normal controls. The LC-MS/MS analysis revealed that these metabolites can be used for the early diagnosis and evaluation of diet therapy.

## 1 Introduction

**Fig. 1:**
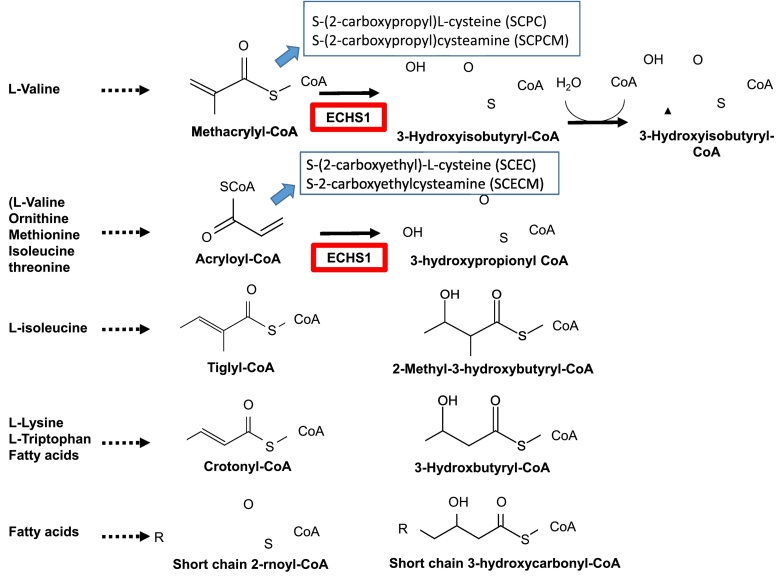
Function of short-chain enoyl-CoA hydratase (ECHS1) and metabolites. Short-chain enoyl-CoA hydratase (ECHS1) is involved in the metabolic pathways of valine, ornithine, methionine, isoleucine, threonine, lysine, tryptophan, and fatty acids. ECHS1 catalyzes the conversion of methacrylyl-CoA, acryloyl-CoA, tiglyl-CoA, crotonyl-CoA, and short-chain 2-rnoyl-CoA to 3-Hydroxyisobutyryl-CoA, 3-hydroxypropionyl CoA, 2-Methyl-3-hydroxybutyryl-CoA, 3-Hydroxbutyryl-CoA, and short-chain 3-hydroxycarbonyl-CoA, respectively. The defect of ECHS1 results in the accumulation of biologically active intermediate metabolites, including four cysteine/cysteamine conjugates from valine metabolites as follows: S-(2-carboxypropyl)cysteine (SCPC) and S-(2-carboxypropyl)cysteamine (SCPCM) from methacrylyl-CoA and S-(2-carboxyethyl)cysteine (SCEC) and S-(2-carboxyethyl)cysteamine (SCECM) from acryloyl-CoA.

Short-chain enoyl-CoA hydratase (ECHS1) is a mitochondrial matrix enzyme that hydrates the double bond of acyl-CoA [1]. This enzyme acts on the metabolic degradation of several amino acids, including valine and fatty acid hydrolysis in mitochondria (Fig. 1) [1], [2], [3], [4]. ECHS1 deficiency (OMIM 602292) causes Leigh syndrome and/or exercise-induced dystonia in milder forms and more than 60 cases have been reported [5], [6], [7]. Patients with ECHS1 deficiency typically present with developmental delay and neurological deterioration from the neonatal or early infantile period, presenting features of Leigh syndrome with basal ganglia lesions and lactic acidemia [8, 9, 10, 11].

ECHS1 is involved in the metabolic pathways of valine, ornithine, methionine, isoleucine, threonine, lysine, tryptophan, and fatty acids. ECHS1 catalyzes the conversion of methacrylyl-CoA, acryloyl-CoA, tiglyl-CoA, crotonyl-CoA, and short-chain 2-rnoyl-CoA to 3-Hydroxyisobutyryl-CoA, 3-hydroxypropionyl CoA, 2-Methyl-3-hydroxybutyryl-CoA, 3-Hydroxbutyryl-CoA, and short-chain 3-hydroxycarbonyl-CoA, respectively. The defect of ECHS1 results in the accumulation of biologically active intermediate metabolites, including four cysteine/cysteamine conjugates from valine metabolites as follows: S-(2-carboxypropyl) cysteine (SCPC) and S-(2-carboxypropyl) cysteamine (SCPCM) from methacrylyl-CoA and S-(2-carboxyethyl) cysteine (SCEC) and S-(2-carboxyethyl) cysteamine (SCECM) from acryloyl-CoA.

ECHS1 has a wide specificity for substrates such as methacrylyl-CoA (valine pathway), acryloyl-CoA (secondary pathway of valine, ornithine, methionine, isoleucine, and threonine), tiglyl-CoA (isoleucine), and crotonyl-CoA (lysine, tryptophan, and fatty acid) (Fig. 1). The defects in 3-hydroxyisobutyryl-CoA hydrolase deficiency presented a Leigh-like phenotype, and blockage of the valine degradation pathway has been regarded as the main pathway responsible for the central nervous system pathology (Fig. 1) [12]. The previous quantitative analyses of patients with ECHS1 deficiency suggest that the accumulation of cysteine and cysteamine conjugates from valine intermediate to methacrylyl-CoA and acryloyl-CoA are responsible for the neuronal cytotoxicity, leading to basal ganglia lesions [11, [9], [13]. Therefore, we measured the following four cysteine/cysteamine conjugates from valine metabolites: S-(2-carboxypropyl) cysteine (SCPC) and S-(2-carboxypropyl) cysteamine (SCPCM) from methacrylyl-CoA and S-(2-carboxyethyl)cysteine (SCEC) and S-(2-carboxyethyl)cysteamine (SCECM) from acryloyl-CoA in urine and serum from six patients (four new patients and two patients reported with ECHS1 deficiency [14]). Moreover, we also speculated the clinical course of the two patients who received the diet therapy. We propose that urine analysis for these cysteine/cysteamine conjugates is useful for the early diagnosis of ECHS1 deficiency.

## 2 Materials and methods

### 2.1 Patients

Six patients with ECHS1 deficiency were included in the valine metabolite analysis, including four newly diagnosed patients (Table 1). The patients included three males and three females, whose ages ranged 1–15 years. Disease onset ranged from 0 to 10 months and the initial symptoms were seizure, nystagmus, liver dysfunction, or neurological regression. Four patients received a diet therapy. Cases three and four have already been reported in a previous study [14] Control urine samples were obtained from six healthy volunteers (5–30 years; mean age 11.3 years, three males and three females).

**Table 1:** Profile of patients with ECHS1 deficiency.

Patient No.1	1	2	3[14]	4[14]	5	6
Sex	M	M	F	M	F	F
Current age (y)	4	7	6	3	15	8
Onset (m)	9	10	10	7	3	8
*ECHS1* variants	c.832G > A, p.(A278T) /?	c.2 T > C, p.(M1?) /c.5C > T, p.(A2V)	c.5C > T, p.(A2V) /c.176A > G, p.(N59S)	same as case 3	c.5C > T, p.(A2V) /c.176A > G, p.(N59S)	same as Case 5
First symptoms	Convulsion, regression	Regression	Nystagmus, regression	Nystagmus, regression	Nystagmus	Convulsion
Head control	+	+	−	−	+	+
Sitting alone	−	−	−	−	+	+
Walk alone	−	−	−	−	−	−
Deterioration	+	−	+	+	+	+
Convulsion	+	+	+	−	+	+
Spastic palsy	+	+	−	−	+	+
Dystonia	+	+	−	−	+	+
Apnea	+	+	−	−	−	−
Liver dysfunction	−	−	−	−	−	−
Lactic acidosis	+	+	−	−		
Diet therapy	Protein restriction	Protein restriction	Valine-removed	Valine removed	N.D.	N.D.
Improvements	Apnea, dystonia	Apnea, dystonia	No change	Development		

### 2.2 Methods

#### 2.2.1 Analysis of metabolites in urine

##### 2.2.1.1 Chemicals and reagents

Acetonitrile and formic acid were obtained from Fujifilm Wako Pure Chemical Co. (Tokyo, Japan). S-(2-carboxypropyl)-cysteine and S-(2-carboxypropyl)-cysteamine were purchased from Angene International Limited (London, UK). S-(2-carboxyethyl)-cysteine, S-(2-carboxypropyl) D2-cysteine, and S-(D3–2-carboxyethyl)-cysteine were chemically produced by Tokyo Chemical Industry (Tokyo, Japan). S-(2-carboxyethyl)-cysteamine was obtained from Sigma-Aldrich (St. Louis, MO, USA).

#### 2.2.2 LC-MS/MS metabolite analysis

Our analytical method was modified from a previous report [11]. Thereafter, 50 μL of urine or serum samples were mixed with 50 μL of 50% acetonitrile/water containing an internal standard and further diluted with water (urine 1:50, serum 1:10). Liquid chromatography with tandem mass spectrometry (LC-MS/MS) analysis was performed using an LCMS-8060 instrument (Shimadzu, Kyoto, Japan). The discovery HS F5–3 column (10 cm × 2.1 mm, 3 μm, Sigma-Aldrich) was maintained at 40 °C. The mobile phases were 0.1% formic acid in water (mobile phase A) and 0.1% formic acid in acetonitrile (mobile phase B). The gradient started from 2% B and was held for 1.4 min. It was increased to 30% B in 6.1 min, increased to 50% B in 2.5 min and held for 2 min, and decreased to 2% B in 0.1 min and held for 3 min. The total run time was 15 min. The flow rate was 0.35 mL/min with an injection volume of 1 μL and 5 μL for urine and serum, respectively. The interface temperature was 300 °C, and the desolvation line and heat block temperatures were 250 °C and 400 °C, respectively. The nebulizing gas flow, heating gas flow, and drying gas flow were 3 L/min, 10 L/min, and 10 L/min, respectively. Samples were kept at 4 °C prior to injection by the autosampler. Multiple reaction monitoring in positive ion mode was used for compound detection. Transitions used for quantification were: S-(2-carboxypropyl)-cysteine, 208.00 > 119.20; S-(2-carboxypropyl)-cysteamine, 164.15 > 119.05; S-(2-carboxyethyl)-cysteine, 194.00 > 105.20; S-(2-carboxyethyl)-cysteamine, 150.10 > 105.05; S-(D3-2-carboxyethyl)-cysteine, 196.95 > 108.10; S-(2-carboxypropyl) D2-cysteine, 209.95 > 119.00. S-(2-carboxypropyl) D2-cysteine was used as a surrogate internal standard for S-(2-carboxypropyl)-cysteamine and S-(2-carboxyethyl)-cysteamine.

#### 2.2.3 ECHS1 activity in the fibroblasts

The fibroblast cells were grown to 70%–80% confluence for approximately two weeks and experiments were performed on cells pooled from the lines. The fibroblasts were purchased from JCRB Cell Bank (Osaka, Japan). The cells were harvested, collected, washed twice with phosphate buffered saline (PBS), and solubilized with 0.5 mL of the lysis buffer containing 25 mM potassium phosphate (pH 8), 2 mM EDTA, 0.1% (*w*/*v*) Triton X-100, and 1:1000-diluted protease inhibitor cocktail (Merck, Darmstadt, Germany). The supernatant was collected after centrifugation at 14,000 rpm for 20 min at 4 °C. The assay conditions for ECHS1 activity were as follows: 100 mM potassium phosphate (pH 8), 0.1 mg/mL bovine serum albumin, 5,5′-dithiobis-(2-nitrobenzoic acid) (DTNB), and 30 μM crotonyl-CoA [10], [15]. Samples were measured at 37 °C in UV cuvettes at 263 nm using a Shimadzu spectrophotometer. The enzymatic activity was normalized to the mitochondrial marker enzyme citrate synthase (CS). CS activity was determined by measuring the reduction of DTNB at 412 nm, coupled with the reduction of acetyl-CoA by citrate synthase in the presence of oxaloacetate. The reaction mixture consisted of 100 mM potassium phosphate (pH 7.5), 0.1 mM DTNB, 0.1 mM acetyl-CoA, and 0.2 mM oxaloacetate. All the reactions were measured for 0.5–2.5 min after adding each substrate. All the reactions were performed in at least three independent experiments in triplicate with similar findings. Enzymatic activity was calculated as mU/mg (1 enzyme unit = the enzyme activity that catalyzes the conversion of 1 μmol substrate into product in 1 min), using a molar extinction coefficient of 6700 M^−1^·cm^−1^ for ECHS1 activity and 13,600 M^−1^·cm^−1^ cm for CS activity and expressed as a percentage of control activity.

#### 2.2.4 Western blot

Whole-cell lysates were prepared in lysis buffer containing 25 mM potassium phosphate (pH 8), 2 mM EDTA, 0.1% (*w*/*v*) Triton X-100, and 1:1000-diluted Protease Inhibitor Cocktail. The lysate was centrifuged at 14,000 rpm for 20 min at 4 °C, the soluble proteins were isolated from the supernatant, and the protein concentration was determined using the Qubit Protein Assay kit (Thermo Fisher Scientific, MA, USA).

Samples were mixed with an equal volume of 2× sodium dodecyl-sulfate polyacrylamide gel electrophoresis (SDS-PAGE) sample loading buffer (0.5 M Tris-HCl, pH 6.8, 10% SDS, 50% glycerol, 2% β-mercaptoethanol, and 5% bromophenol blue). Protein samples were separated on a 4%–12% gradient SDS-polyacrylamide mini-gel. Proteins were electrophoretically transferred onto polyvinylidene difluoride membranes. Membranes were blocked in 5% PBS and incubated overnight with primary antibodies, rabbit anti-ECHS1 from Abcam (OR, USA), and mouse anti-beta-actin from Sigma-Aldrich (MO, USA). Following three washes with PBS-0.05% Tween 20, the membranes were incubated with horseradish peroxidase-conjugated secondary antibody (Santa Cruz, TX, USA) diluted in 5% PBS. The secondary antibody was detected using the chemiluminescence ECL Plus reagent (Takara Bio, Shiga, Japan) and the membranes were visualized using the Image Quant LAS4000 Imaging System (GE Healthcare, IL, USA).

#### 2.2.5 Genetic analysis

DNA was extracted from the whole blood samples using a ReliaPrep-Blood-gDNA-Miniprep-System (Promega, Madison, WI, USA). Whole exome sequences were obtained from Riken Genetics (Osaki, Tokyo, Japan). For Sanger sequencing, primers were designed for polymerase chain reaction (PCR) amplification (9700, Applied Biosystems, MA, USA) of the ECHS1 gene (forward primer for exon 4, 5′-tggcagcagagcctgtaaga-3′; reverse primer for exon 4, 5′-tgagacacaggcagattttgag-3′; forward primer for exon 8, 5′-agctctgattgggcaggtgt-3′; and reverse primer for exon 8, 5′- attggagaggaactgcacacc-3′). PCR was performed using the KOD FX Neo DNA polymerase kit (Toyobo Inc. Osaka, Japan) with 25 μL of the reaction mixture according to the manufacturer's instructions. The PCR conditions were as follows: initial denaturation at 94 °C for 2 min, one cycle; 98 °C for 10 s, 64 °C for 30 s, 40 cycles; and final extension at 72 °C for 7 min, one cycle. The PCR products were electrophoresed on a 2% agarose gel and visualized with ethidium bromide. The amplification products were directly sequenced using an ABI 3730 sequencer (Applied Biosystems, MA, USA).

#### 2.2.6 Diet therapy

Two types of treatment milk (Diet therapy 1 and 2) were prepared to reduce the special amino acids (Supplementary Table 1).

##### 2.2.6.1 Diet therapy 1. Protein restriction milk

Protein-removed milk (S-23, Yukijirushi, Tokyo, Japan) and enteric nutrients (RACOL® NF liquid, EN Otsuka Pharmaceutical Co. Ltd. Hanamaki) were equally mixed. The ingredients used are listed in Supplementary Table 1. The total amount of milk was 800 mL (calories: 58 kcal/kg/day and protein: 1.2 g/kg /day). Valine intake was limited to 61 mg/kg/day.

##### 2.2.6.2 Diet therapy 2. Valine-leucine-isoleucine-lysine-tryptophan restricted milk

The second diet treatment aimed to limit the intake of valine (70 mg/kg/day), leucine (90 mg/kg/day), and isoleucine (50 mg/kg/day). Valine-leucine-isoleucine-removed milk (S-22, Yukijirushi, Tokyo) used for disorders affecting the valine catabolic pathway and lysine-tryptophan-removed milk used for glutaric acidemia type 1 (S-30, Yukijirushi, Tokyo) were equally mixed. The ingredients used are listed in Supplementary Table 1. The total amount of milk was 800 mL (calories: 68.9 kcal/kg/day and protein: 1.9 g/kg /day).

## 3 Results

### 3.1 Clinical profile of patients with ECHS1 deficiency (Table 1)

#### 3.1.1 Measurement of substrates of ECHS1, internal metabolites of valine in serum and urine. (Fig. 2 and Supplementary Table 2)

**Fig. 2:**
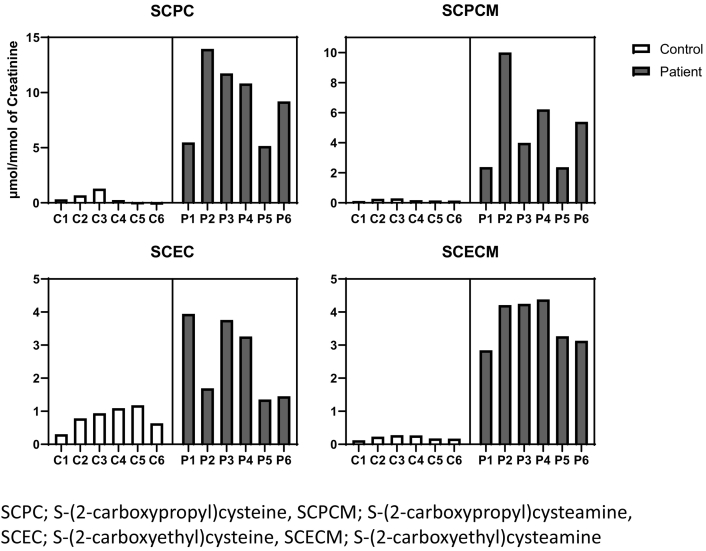
Urine level of cysteine/cysteamine conjugates. The levels of four cysteine/cysteamine conjugates: we measure four cysteine/cysteamine conjugates from valine metabolites as follows: S-(2-carboxypropyl)cysteine (SCPC) and S-(2-carboxypropyl)cysteamine (SCPCM) from methacrylyl-CoA and S-(2-carboxyethyl)cysteine (SCEC) and S-(2-carboxyethyl)cysteamine (SCECM) from acryloyl-CoA in urine and serum from six patients.

In ECHS1 deficiency, valine intermediate metabolites, methacrylyl-CoA and acryloyl-CoA, were reportedly elevated [11], [15]. SCPC, SCPCM, SCEC, and SCECM were measured using LC-MS/MS in urine and serum samples. Urine levels of these four metabolites were elevated in all the patients (*p* = 0.002 for SCPC, 0.009 for SCPCM, 0.014 for SCEC, and 0.00008 for SCECM, respectively: Fig. 2 and Supplementary Table 2).

Serum metabolites were analyzed in three controls and two patients. The levels of these four metabolites were not significantly different (Supplementary Fig. 2 and Supplementary Table 3).

### 3.2 Clinical course of two patients received the diet therapy

#### 3.2.1 Patient 1

**Fig. 3:**
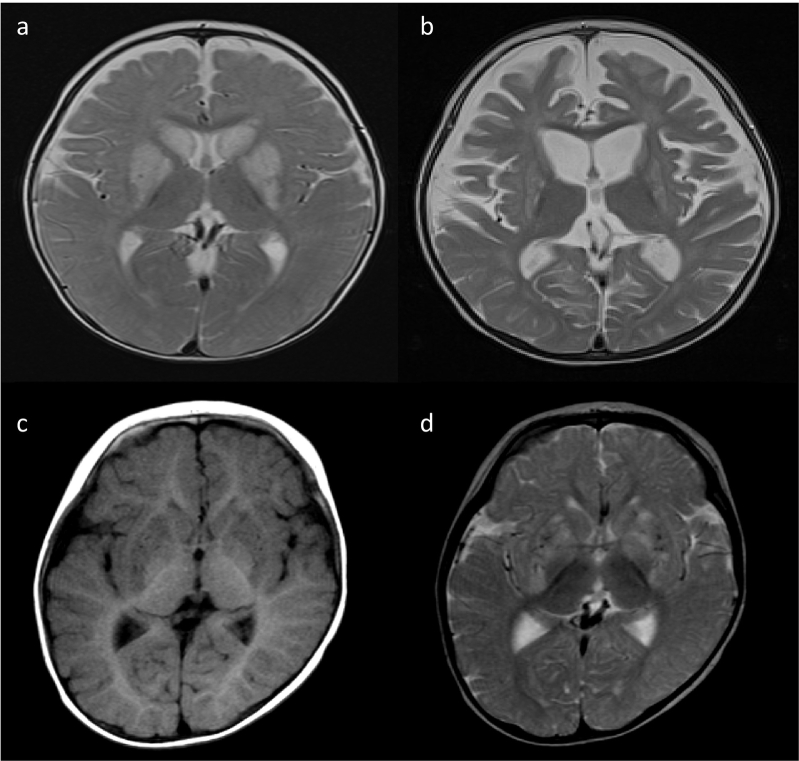
MRI findings. High intensity is observed in the striatum and globus pallidus on T2-weighted images (T2WI) at 8 months of age: (a) atrophy of the cerebral cortex and basal ganglia is observed on T2WI at 19 months (b) in patient 1. Low intensity is observed in the putamen, globus pallidus, and caudate nucleus on T1-weighted images (T1WI) at 10 months of age (c) and high intensity is observed in the putamen, globus pallidus, and caudate nucleus on T2WI at 10 months of age (d) in patient 2.

**Fig. 4:**
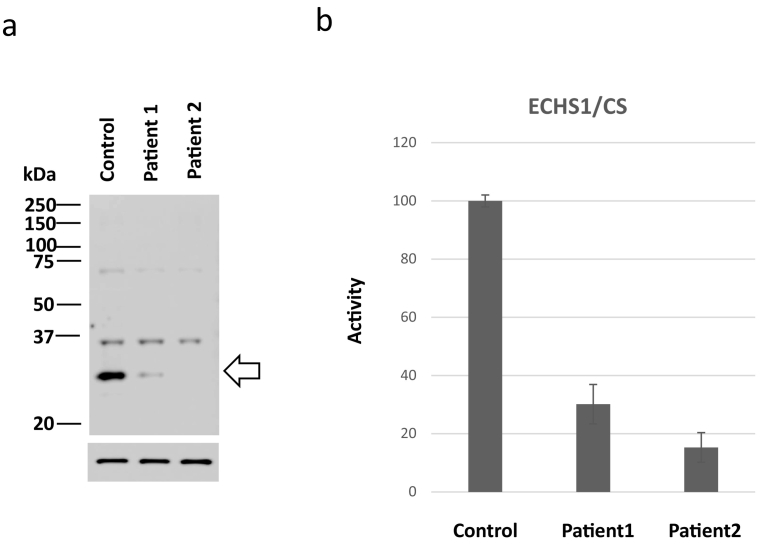
Expression and activity of short-chain enoyl-CoA hydratase (ECHS1) in fibroblasts of patient 1. a) Expression of short-chain enoyl-CoA hydratase (ECHS1) (allow, 31 kDa), faint in patient 1(lane 2), is not detected in patient 2 (lane 3). The expression of beta-actin (allow head, 42 kDa) is the same in both control and the patients.

Patient 1 was a three-year-old boy. His parents were healthy and unrelated. He was delivered at 40th week of gestation. His birth weight was 2980 g. He had gained head control at the age of four months and could roll over at the age of seven months albeit not sit. At the age of eight months, he presented status epilepticus and exhibited spasticity, dystonia, apnea, and deterioration to bedridden. He was administered anti-epileptic drugs and muscle relaxants. His clinical course and metabolic analysis were performed during the diet therapy. His lactate and pyruvate levels in CSF were 21.3 mg/dL and 1.7 mg/dL, respectively. Urine organic analysis presented 2,3-OH-2-methylbutyric acid peak, and magnetic resonance imaging (MRI) presented a high intensity in the caudate and globus pallidus on T2-weighted image (T2WI) at the age of 8 months (Fig. 3a) and diffuse atrophy of the cortex and basal ganglia at the age of 19 months (Fig. 3b). He was diagnosed with Leigh syndrome. Whole exome sequencing (WES) presented no genomic variants that accounted for Leigh syndrome, except heterozygous mutations in maternal c.832G > A, p.(Ala278Thr) in *ECHS1*. In silico analysis predicted this variant to be pathogenic (Supplementary Table 4: SIFT, 0; Polyphen-2, 0.985; CADD, 23; REVEL, 0.389; Mutation Assessor, 0.938; and Mutation Taster, 0.999). Sanger sequencing confirmed this genomic variant, however, no variant was detected in the other allele. Western blotting demonstrated a faint expression of ECHS1 (Fig. 4a), and ECHS1 activity of the fibroblasts was 30.2% of that of the control (Fig. 4b). The patient was diagnosed with Leigh syndrome secondary to possible ECHS1 deficiency.

**Fig. 5:**
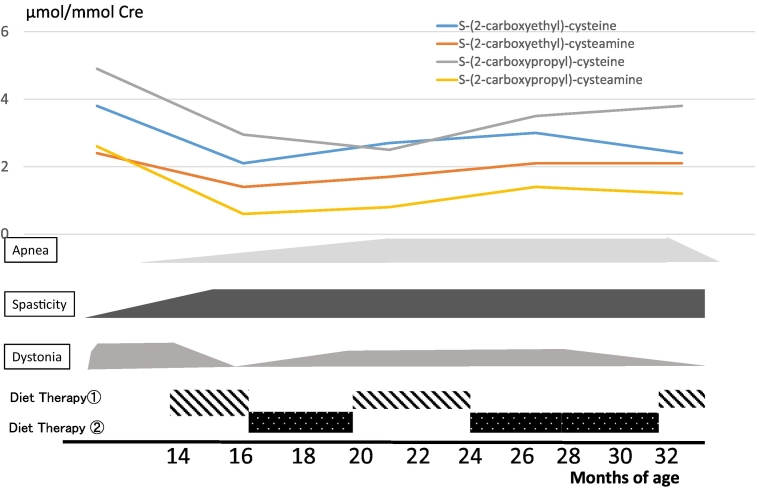
Clinical course of patient 1 during diet therapy. Changes in the urinary levels of valine metabolites and symptoms are observed during the diet therapy in patient 1. Sixteen months following the first diet therapy 1, the patient's condition improves such that he has visual fixation and follows objects and dystonia disappears. Following the third diet therapy 1, apnea disappears However, spasticity remains; visual fixation is gradually lost, regardless of diet. Metabolic analysis presents improved levels of the toxic internal metabolites of valine. In contrast, diet therapy 2 does not improve the symptoms and the level of metabolites increases.

Diet therapy 1 was administered from 14 to 16 months, 20 to 24 months, and post 32 months; diet therapy 2 was administered from 16 to 20 months and 24 to 32 months (Fig. 5). Following the first diet therapy 1, the patient's neurological condition improved such that he had visual fixation and followed objects and dystonia disappeared at 16th month. Metabolic analysis presented an improved level of the toxic intermediate metabolites of valine. Thereafter, when we tried diet therapy 2, dystonia gradually reappeared and apnea increased. Hence, we returned to diet therapy 1. The patient's condition did not improve, therefore, we repeated diet therapy 2. Even following the second diet therapy 2, the patient's condition did not improve and the metabolites were elevated compared with those in diet therapy 1. We returned to diet therapy 1. Following the third diet therapy 1, apnea disappeared. However, spasticity remained and visual fixation was gradually lost, regardless of the diet therapy. (Fig. 5a).

#### 3.2.2 Patient 2

Patient 2 was a seven-year-old boy. His parents were healthy and unrelated. He was delivered at 40th week of gestation. His birth weight was 3976 g. He had gained head control at the age of four months and could sit for eight months. At 10th month, he lost consciousness following fever and five days of diarrhea, without biochemical abnormalities such as metabolic acidosis, hypoglycemia, lactic acidosis, and CSF lactate elevation. The urine organic acid analysis results were normal. MRI indicated a low intensity on the bilateral striatum and caudate nucleus on T1-weighted image (T1WI) (Fig. 3c) and high intensity on T2WI (Fig. 3d). Further, he deteriorated to bedridden. However, he gradually improved to roll over and eat orally with a complete support. He needed anti-epileptic drugs owning to focal epileptic seizures as sequelae.

**Fig. 6:**
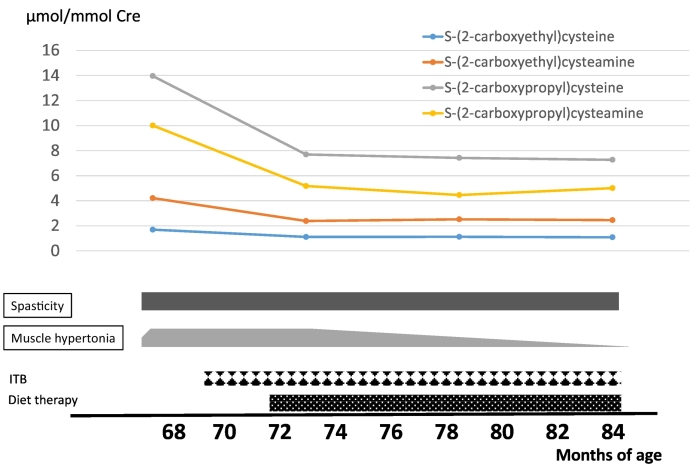
Clinical course of patient 2 during the diet therapy. In patient 2, diet therapy 1 is administered. Following the diet therapy, hypotonia improves and the levels of metabolites, especially S-(2-carboxypropyl)cysteine (SCPC) and S-(2-carboxypropyl)cysteamine (SCPCM) decrease.

At the age of 13 months, he lost consciousness with high fever, ketoacidosis, and lactic acidosis. Serum lactate/pyruvate levels were elevated to 3.53/0.295 mmol/L and CSF lactate/pyruvate levels were 4.88/0.37 mmol/L, respectively. A brain MRI indicated a high-intensity signal in the bilateral striatum on T2WI and diffused-weighted imaging. In addition, MR spectroscopy revealed a lactate signal in the putamen (Supplementary Fig. 3). Following repeated encephalopathy triggered by viral infections, the patient's muscle hypertonia, spasticity, and dystonia were exacerbated and uncontrolled. These symptoms improved slightly with intrathecal baclofen therapy at four years of age. He was diagnosed with ECHS1 deficiency based on genetic and biochemical analyses. Compound heterozygous variants, c.2 T > C, p. (Met1?) / c.5C > T p. (Ala2Val) were identified in *ECHS1* (NM_004092) by WES. Silico analysis predicted p. (Ala2Val) as pathogenic and p. (Met1?) as uncertain. Western blotting demonstrated no expression of ECHS1 (Fig. 4a), and ECHS1 activity of fibroblasts was ~15% of that of the control (Fig. 4). The patient was diagnosed with Leigh syndrome due to ECHS1 deficiency. His clinical course and metabolic analysis were performed during the diet therapy (Fig. 6). During diet therapy 1 with a combination of half protein-free milk and half parenteral nutrition (RACOL-NF Liquid for Enteral Use), his muscle hypertonia, spasticity, and dystonia were markedly improved. Urinary valine metabolite levels decreased following protein restriction (Fig. 6).

## 4 Discussion

This study aimed to establish the diagnostic methods for ECHS1 deficiency using urine and serum samples of the patients. Therefore, we measured the following four cysteine/cysteamine conjugates: SCEC, SCECM, SCPC, and SCPCM. Several metabolites have been reportedly elevated in patients with ECHS1 deficiency and urine tandem mass screening has been implicated as a screening method [11], [16]. However, the diagnostic value of these metabolites has not yet been established, since some cases with mild phenotypes have failed to show the elevation [15]. We examined the diagnostic value of the four metabolites for the diagnosis of ECHS1. Among them, the urinary levels of SCPC, SCPCM, and SCECM were significantly elevated in patients with ECHS1 deficiency compared to controls (Fig. 2, Table 2). SCEC was not markedly elevated compared with that in some controls. Therefore, the urine levels of SCPC, SCPCM, and SCECM could be used to diagnose ECHS1 deficiency. Moreover, the serum levels of these metabolites were not different between normal controls and patients and appeared unfeasible for screening of ECHS1 deficiency.

SCPC and SCPCM are valine metabolites that accumulate from the defects of methacrylyl-CoA to (*S*)-3-hydroxyisobutyryl-CoA and have been considered to induce the cell toxicity and relate to the clinical severity [11, [9], [13]. The levels of urinary SCPC and SCPCM in patient 5, who was the mildest to roll over and sit with support were the lowest among all the patients. The levels of these two metabolites may correlate with the disease severity.

The effect of diet therapy has been reported in several patients with ECHS1 deficiency [8], [17], [18], [19]. In the cases of brothers, case 4 initiated restriction therapy at the age of 14 months and stayed healthier than the younger brother, who started the diet therapy at the age of four years (Table 1) [14]. Abdenur reported the clinical course post a valine restriction diet for three cases with ECHS1 deficiency [19]. All three cases presented improved awareness, interaction, and dystonia [19].

Case 1 was administered two diet therapy recipes as follows: protein-restricted diet (diet 1) and valine-leucine-isoleucine-lysine-tryptophan (diet 2). Diet therapy 1 improved some symptoms, such as dystonia in patient 1 and hypotonia in patient 2. Diet therapy 2 did not improve the symptoms or level of metabolites. The reasons for the difference in the effects of these two diets are not completely understood, since the amount of valine in diet 1 (129 mg/kg/day) was higher than in diet 2 (75 mg/kg/day). The other amino acid composition may have contributed to these effects. Timing and the age of therapy may be related to these effects. As we have checked only one urine sample at pre-diet, we cannot exclude the possibility that the metabolite response in Case 1 is within normal variation. Case 3 was also administered protein-restricted milk and presented a decrease in hypertonicity. The further diet therapy and cell toxicity studies for valine metabolites and other metabolites will contribute to the development of an optimal diet for ECHS1.

In conclusion, we presented that the urinary levels of SCPC, SCPCM, and SCECM can be used to diagnose ECHS1 deficiency. An early diagnosis of ECHS and protein restriction therapy may alleviate the symptoms of ECHS1 deficiency.

The following are the supplementary data related to this article.

**Supplementary Fig. 1. Position of mutations in *ECHS1***
Positions of new mutations found in patients 1 and 2 UTR, untranslated regions; CDS, coding sequences.
**Supplementary Fig. 2. Serum level of cysteine/cysteamine conjugates**
Levels of four cysteine/cysteamine conjugates. We measured four cysteine/cysteamine conjugates to valine metabolites; S-(2-carboxypropyl) cysteine (SCPC) and S-(2-carboxypropyl) cysteamine (SCPCM) from methacrylyl-CoA, and S-(2-carboxyethyl) cysteine (SCEC), and S-(2-carboxyethyl) cysteamine (SCECM) from acryloyl-CoA in urine and serum from 4 controls and 2 patients.
**Supplementary Fig. 3. MR spectroscopy in patient 2**
MR spectroscopy shows lactate peak reflecting high lactate level in the CNS. Image 1

Supplementary Table 1 Ingredients of various diets. Supplementary Table 1

Supplementary Table 2 Urine levels of SCPC, SCPCM, SCEC, and SCECM. Supplementary Table 2

Supplementary Table 3 Serum levels of SCPC, SCPCM, SCEC, and SCECM. Supplementary Table 3

Supplementary Table 4 In silico analysis of variants. Supplementary Table 4

## Ethical statement

This study was performed in accordance with the ethical standards of the Declaration of Helsinki and approved by the Ethics Committee of Jichi Medical School 2017.

Informed consent was obtained from the parents of all the patients for the investigatory work.

## Author statement

None.

## Declaration of Competing Interest

Authors report no conflicts of interest.

b) Activity of ECHS1 is measured by the level of crotonyl-CoA following the addition of lysine.

The ECHS1 enzyme activities of skin fibroblasts are 30.2% (patient 1) and 15.2% (patient 2) of the control (100%).
