# Supplementary material for: Valine metabolites analysis in ECHS1 deficiency
Source: Mol Genet Metab Rep. 2021 Oct 9;29:100809. doi: 10.1016/j.ymgmr.2021.100809 (PMC8507190; doi:10.1016/j.ymgmr.2021.100809)
Supplement: Supplementary file 1 — Supplementary Fig. 1. Position of mutations in ECHS1 Positions of new mutations found in patients 1 and 2 UTR, untranslated regions; CDS, coding sequences. Supplementary Fig. 2. Serum level of cysteine/cysteamine conjugates Levels of four cysteine/cysteamine conjugates. We measured four cysteine/cysteamine conjugates to valine metabolites; S-(2-carboxypropyl) cysteine (SCPC) and S-(2-carboxypropyl) cysteamine (SCPCM) from methacrylyl-CoA, and S-(2-carboxyethyl) cysteine (SCEC), and S-(2-carboxyethyl) cysteamine (SCECM) from acryloyl-CoA in urine and serum from 4 controls and 2 patients. Supplementary Fig. 3. MR spectroscopy in patient 2 MR spectroscopy shows lactate peak reflecting high lactate level in the CNS. [file mmc1.pptx]

## Slide 1
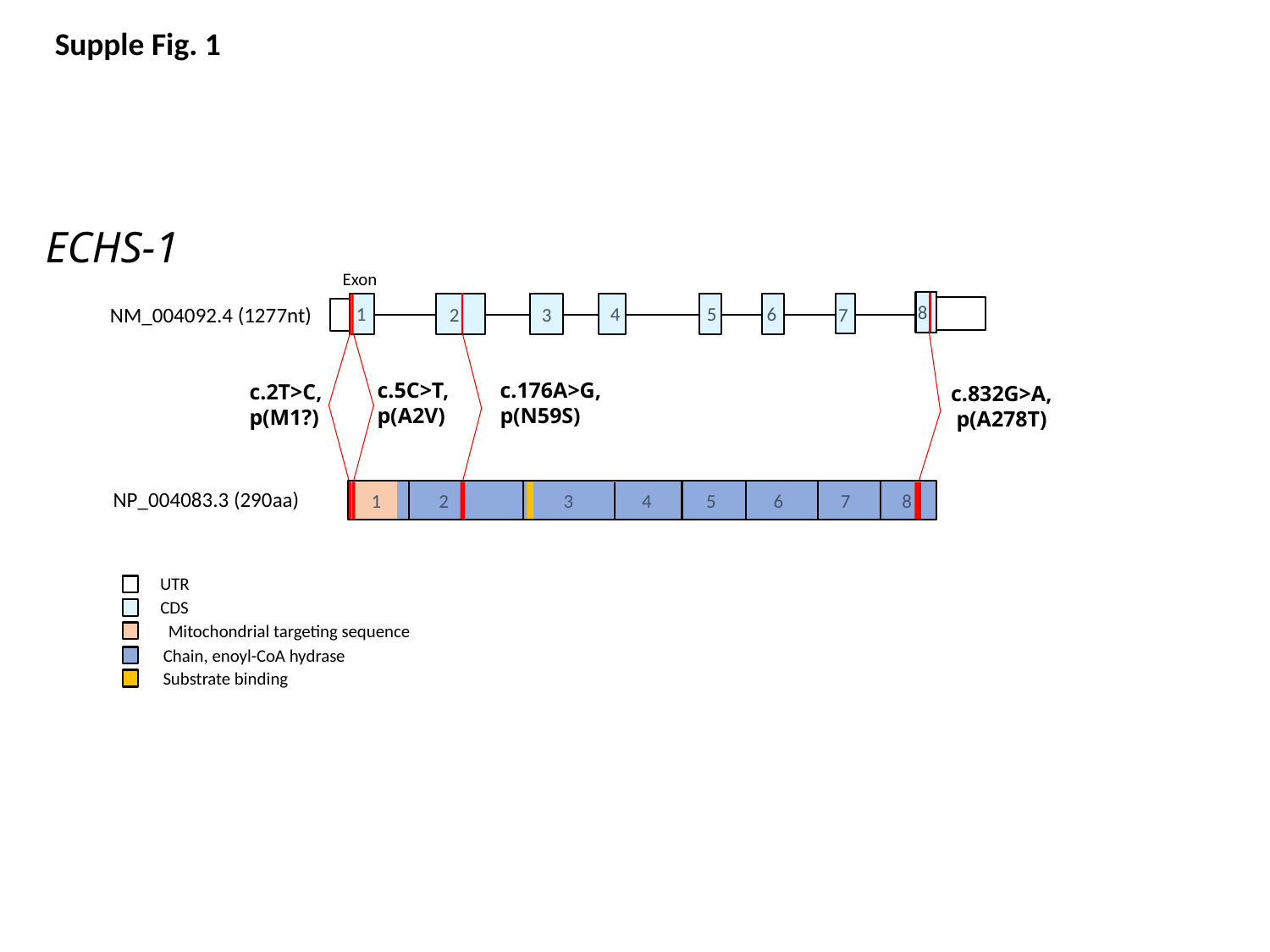

Supple Fig. 1
ECHS-1
Exon
8
NM_004092.4 (1277nt)
5
6
1
4
3
7
2
c.5C>T,
p(A2V)
c.176A>G,
p(N59S)
c.2T>C,
p(M1?)
c.832G>A,
 p(A278T)
NP_004083.3 (290aa)
1
2
3
4
5
6
7
8
UTR
CDS
Mitochondrial targeting sequence
Chain, enoyl-CoA hydrase
Substrate binding

## Slide 2
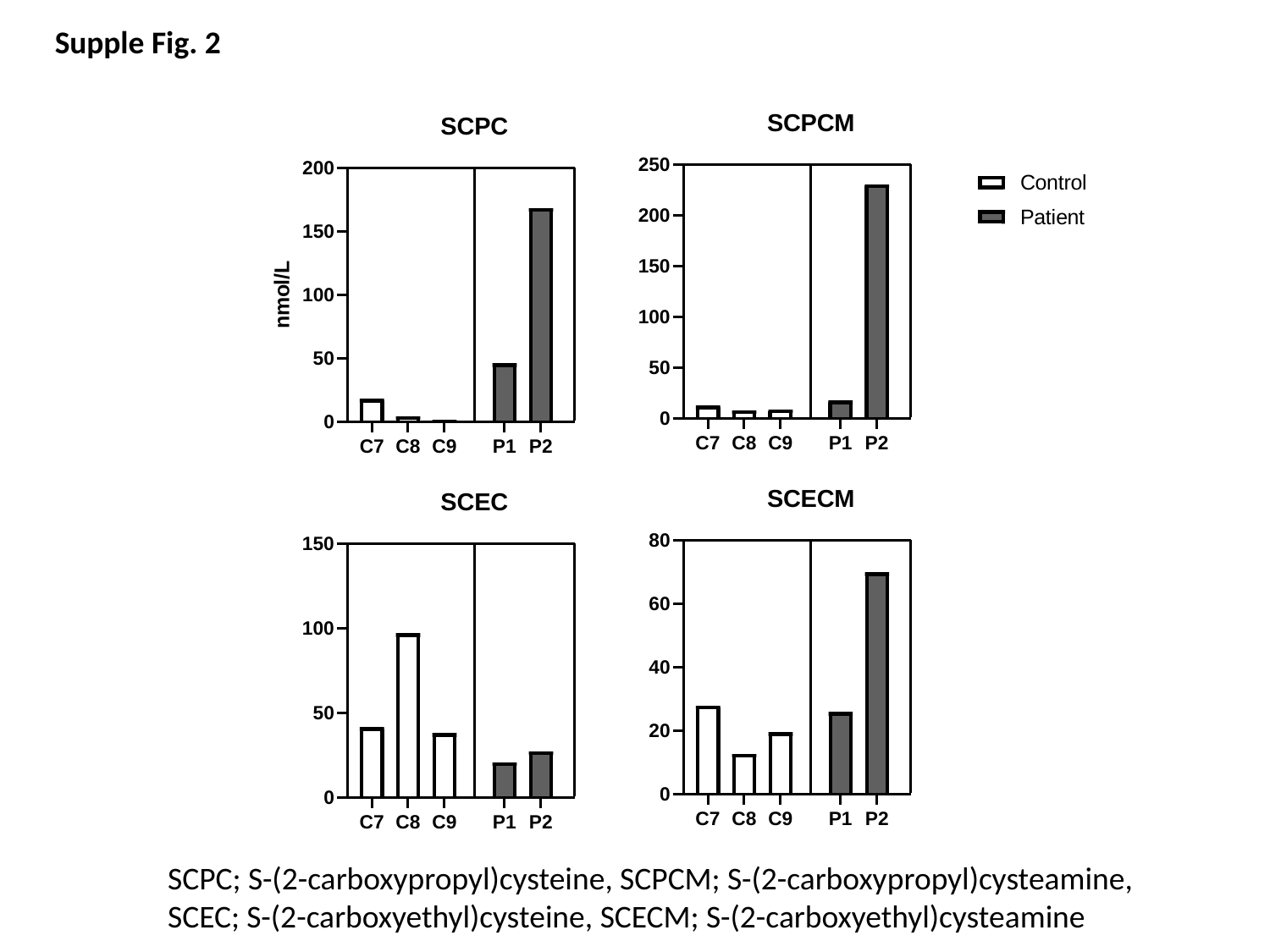

Supple Fig. 2
SCPC; S-(2-carboxypropyl)cysteine, SCPCM; S-(2-carboxypropyl)cysteamine,
SCEC; S-(2-carboxyethyl)cysteine, SCECM; S-(2-carboxyethyl)cysteamine

## Slide 3
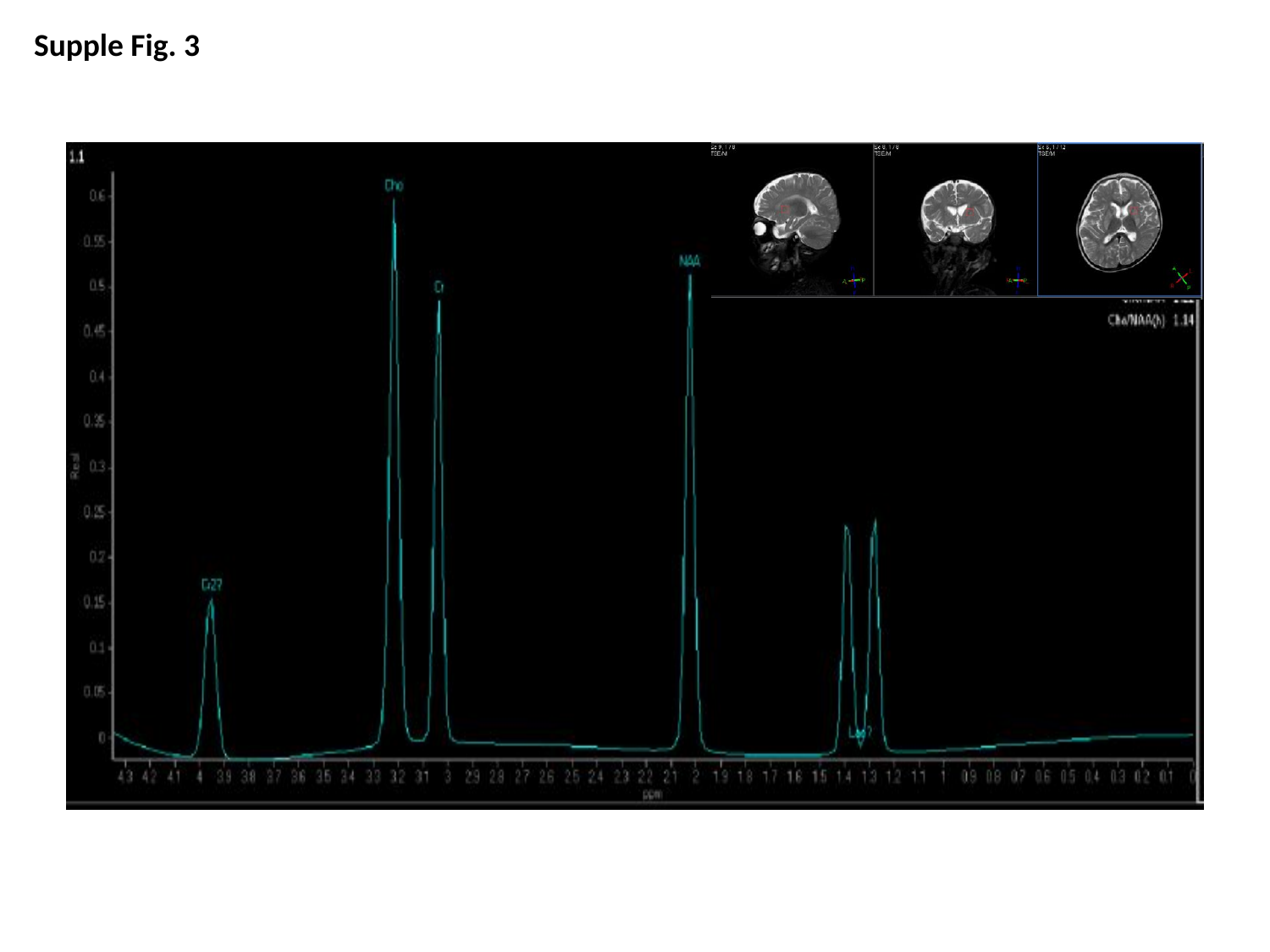

Supple Fig. 3
