# Supplementary material for: Valine metabolites analysis in ECHS1 deficiency
Source: Mol Genet Metab Rep. 2021 Oct 9;29:100809. doi: 10.1016/j.ymgmr.2021.100809 (PMC8507190; doi:10.1016/j.ymgmr.2021.100809)
Supplement: Supplementary Table 1 — Ingredients of various diets. [file mmc2.docx]

**Supplementary Table 1**

| Product name |  | **RACOL-NF** | **S-23** | **Diet therapy 1** |  | **S-22** | **S-30** | **Diet therapy 2** |
| --- | --- | --- | --- | --- | --- | --- | --- | --- |
| characteristics |  | Enteric nutrient | Protein-removed milk | RACOL:S-23=1:1 |  | Valine-leucine-isoleucine-removed | Lysine-tryptophan-removed | S-22:S-30=1:1 |
| volume |  | 100 mL | 15% (w/v) 100 mL | 100 mL |  | 15% (w/v) 100 ml | 15% (w/v) 100 ml | 100 mL |
| Protein | G | 4.38 | 0 | 2.19 |  | 1.8 | 2.07 | 1.935 |
| Lipid | G | 2.23 | 3.27 | 2.75 |  | 2.57 | 2.57 | 2.57 |
| Carbohydrate | G | 15.62 | 11 | 13.31 |  | 9.83 | 9.56 | 9.695 |
| Energy | kcal | 100 | 72.5 | 86.25 |  | 68.9 | 68.9 | 68.9 |
| phenylananine | Mg | 226 | 0 | 113 |  | 92 | 90 | 91 |
| isoleucine | Mg | 204 | 0 | 102 |  | 0 | 105 | 52.5 |
| leucine | Mg | 377 | 0 | 188.5 |  | 174 | 174 | 174 |
| valine | Mg | 258 | 0 | 129 |  | 0 | 150 | 75 |
| methionine | Mg | 97 | 0 | 48.5 |  | 0 | 75 | 37.5 |
| threonine | Mg | 172 | 0 | 86 |  | 0 | 68 | 34 |
| tryptophan | Mg | 54 | 0 | 27 |  | 44.4 | 0 | 22.2 |
| lysine | Mg | 280 | 0 | 140 |  | 230 | 0 | 115 |
| histidine | Mg | 129 | 0 | 64.5 |  | 76.5 | 77 | 76.75 |
| arginine | Mg | 183 | 0 | 91.5 |  | 147 | 147 | 147 |
| aspartic acid | Mg | 366 | 0 | 183 |  | 153 | 150 | 151.5 |
| cysteine | Mg | 32 | 0 | 16 |  | 80 | 80 | 80 |
| glutamic acid | Mg | 915 | 0 | 457.5 |  | 299 | 299 | 299 |
| glycine | Mg | 108 | 0 | 54 |  | 0 | 150 | 75 |
| proline | Mg | 387 | 0 | 193.5 |  | 153 | 153 | 153 |
| serine | Mg | 237 | 0 | 118.5 |  | 108 | 108 | 108 |
| tyrosine | Mg | 183 | 0 | 91.5 |  | 92 | 92 | 92 |
| alanine | Mg | 151 | 0 | 75.5 |  | 153 | 153 | 153 |
|  |  |  |  |  |  |  |  |  |
