# Supplementary material for: Valine metabolites analysis in ECHS1 deficiency
Source: Mol Genet Metab Rep. 2021 Oct 9;29:100809. doi: 10.1016/j.ymgmr.2021.100809 (PMC8507190; doi:10.1016/j.ymgmr.2021.100809)
Supplement: Supplementary Table 2 — Urine levels of SCPC, SCPCM, SCEC, and SCECM. [file mmc3.docx]

**Supplementary Table 2**

| Sample | SCPC | SCPCM | SCEC | SCECM |
| --- | --- | --- | --- | --- |
| C1 | 0.32 | 0.12 | 0.31 | 0.12 |
| C2 | 0.68 | 0.27 | 0.78 | 0.23 |
| C3 | 1.29 | 0.30 | 0.94 | 0.28 |
| C4 | 0.26 | 0.18 | 1.09 | 0.27 |
| C5 | 0.08 | 0.16 | 1.18 | 0.18 |
| C6 | 0.06 | 0.15 | 0.63 | 0.17 |
| P1 | 5.47 | 2.38 | 3.94 | 3.01 |
| P2 | 13.96 | 10.01 | 1.69 | 4.25 |
| P3 | 11.73 | 3.99 | 3.76 | 2.84 |
| P4 | 10.83 | 6.22 | 3.26 | 2.38 |
| P5 | 5.16 | 2.37 | 1.36 | 3.27 |
| P6 | 9.20 | 5.39 | 1.45 | 3.13 |
|  | p=0.001 | p=0.009 | p=0.015 | p<0.001 |

Urine levels in µmol/mmol of creatinine.　SCPC: S-(2-carboxypropyl) cysteine, SCPCM: S-(2-carboxypropyl) cysteamine, SCEC: S-(2-carboxyethyl) cysteine, SCECM: S-(2-carboxyethyl) cysteamine
