# Supplementary material for: Valine metabolites analysis in ECHS1 deficiency
Source: Mol Genet Metab Rep. 2021 Oct 9;29:100809. doi: 10.1016/j.ymgmr.2021.100809 (PMC8507190; doi:10.1016/j.ymgmr.2021.100809)
Supplement: Supplementary Table 3 — Serum levels of SCPC, SCPCM, SCEC, and SCECM. [file mmc4.docx]

**Supplementary Table 3**

Sample SCPC SCPCM SCEC SCECM

C7 18.2 12.6 41.6 27.8

C8 4.5 8.2 97.2 12.6

C9 2.0 9.1 38.2 19.5

P1 46.4 17.8 20.8 25.9

P2 168.5 230.6 27.4 69.8

p = 0.35 p = 0.48 p = 0.21 p = 0.42

Serum levels are presented in nmol/L

SCPC: S-(2-carboxypropyl) cysteine, SCPCM: S-(2-carboxypropyl) cysteamine, SCEC: S-(2-carboxyethyl) cysteine, SCECM: S-(2-carboxyethyl) cysteamine
