# Supplementary material for: Valine metabolites analysis in ECHS1 deficiency
Source: Mol Genet Metab Rep. 2021 Oct 9;29:100809. doi: 10.1016/j.ymgmr.2021.100809 (PMC8507190; doi:10.1016/j.ymgmr.2021.100809)
Supplement: Supplementary Table 4 — In silico analysis of variants. [file mmc5.docx]

**Supplementary Table 4**

ClinVar SI​FT Polyphen-2 CADD REVEL Mutation Assessor Mutation Taster

c.832G>A, p.(A278T) Uncertain significance 0 0.985 23 0.389 0.938 0.999

c.2T>C, p.(M1?) No data 0 0.419 22 0.284 - -

c.5C>T, p.(A2V) Pathogenic 0.08 0.011 21 0.604 0.144 0.998

c.176A>G, p.(N59S) Pathogenic 0 0.992 24 0.712 0.98 0.999
